# The Impact of Intra-abdominal Pressure on Perioperative Outcomes in Laparoscopic Cholecystectomy

**DOI:** 10.7759/cureus.71679

**Published:** 2024-10-17

**Authors:** Muhammad Attaullah Khan, Ihtisham Haq, Zain Ihsan, Muhammad Daud, Naveed Ahmad, Hazrat Ali, Farhan Aslam, Sahibzada Saad Ur Rehman

**Affiliations:** 1 Surgical Oncology, Shaukat Khanum Memorial Cancer Hospital and Research Center, Lahore, PAK; 2 Surgical Oncology, Lady Reading Hospital MTI (Medical Teaching Institution), Peshawar, PAK; 3 General Surgery, Lady Reading Hospital MTI (Medical Teaching Institution), Peshawar, PAK

**Keywords:** intra-abdominal pressure, laparoscopic cholecystectomy, operative time, perioperative outcomes, postoperative pain

## Abstract

Introduction: Intra-abdominal pressure (IAP) is a critical factor in laparoscopic cholecystectomy, potentially affecting various perioperative outcomes. Understanding the impact of different IAP levels can guide clinical practice to optimize patient safety and recovery.

Objective: This study aimed to evaluate the effects of low, moderate, and high IAPs on perioperative outcomes in patients undergoing laparoscopic cholecystectomy.

Methodology: A total of 150 patients were categorized into three groups based on IAP: Low Pressure (8-10 mmHg, n=50), Moderate Pressure (10-15 mmHg, n=70), and High Pressure (>15 mmHg, n=30). Primary outcomes measured included operative time, intraoperative complications, postoperative pain scores at six hours, length of hospital stay, and return to normal activity. Secondary outcomes included postoperative complications and analgesic consumption.

Results: Operative time increased significantly with higher IAP, averaging 80.4 ± 12.7 minutes in the Low Pressure group, 85.6 ± 15.3 minutes in the Moderate Pressure group, and 90.1 ± 14.2 minutes in the High Pressure group (p=0.03). Intraoperative complications occurred in 10%, 15%, and 23% of patients in the Low, Moderate, and High Pressure groups, respectively (p=0.12). Postoperative pain scores, length of hospital stay, and recovery time showed no significant differences between groups.

Conclusion: Higher IAP is associated with longer operative times but does not significantly affect other perioperative outcomes, suggesting that careful consideration of IAP levels is important in laparoscopic cholecystectomy.

## Introduction

Laparoscopic cholecystectomy is a commonly performed surgical procedure for the treatment of symptomatic gallstones and is known for its minimal invasiveness compared to traditional open surgery. This technique, characterized by its use of small incisions and a laparoscope, offers benefits such as reduced postoperative pain, shorter hospital stays, and quicker recovery times. Despite its advantages, the procedure's effectiveness and safety can be influenced by various factors, one of the most significant being intra-abdominal pressure (IAP) [1,2]. IAP is the pressure within the abdominal cavity, which is controlled and monitored during laparoscopic surgery using insufflation of carbon dioxide (CO_2_) to create a working space for the surgeon [3].

The role of IAP in laparoscopic surgery is multifaceted. On the one hand, adequate IAP is crucial for creating sufficient space within the abdomen to visualize and manipulate surgical instruments effectively [4]. On the other hand, excessive IAP can lead to complications such as impaired respiratory function, cardiovascular instability, and increased postoperative pain. The balance between achieving an optimal working space and avoiding elevated pressures that could negatively impact patient outcomes is a key concern for surgeons [5].

The impact of IAP on perioperative outcomes in laparoscopic cholecystectomy has been a subject of extensive research. IAP is typically adjusted to a level between 10 and 15 mmHg during the procedure. This pressure range is considered sufficient to provide visibility and access without significantly increasing the risk of complications [6]. However, variations in IAP levels can affect several perioperative parameters, including operative time, postoperative pain, and recovery duration.

One of the primary concerns with higher IAP is its potential effect on operative time. Elevated IAP can lead to increased difficulty in maneuvering surgical instruments and a greater likelihood of accidental injury to surrounding organs. Conversely, too low an IAP may not provide adequate space for effective surgical intervention. Studies have shown that while a moderate increase in IAP may slightly extend operative time, the benefits of improved visibility and reduced tissue interference often outweigh these concerns [7,8].

The level of IAP also plays a critical role in postoperative pain management. Higher pressures can cause more significant stretching of the abdominal wall and diaphragm, leading to increased pain and discomfort after the procedure. Lower IAP levels generally correlate with reduced postoperative pain, though this must be balanced with the need for adequate visibility during surgery [9]. Effective pain management strategies are essential to improve patient comfort and enhance recovery.

Another important aspect influenced by IAP is the length of hospital stay and the time to return to normal activities. Elevated IAP can contribute to longer recovery times and delays in resuming normal activities due to its impact on postoperative pain and overall patient well-being. Conversely, a lower IAP may facilitate faster recovery, though this must be weighed against potential challenges in maintaining surgical visibility [8,9].

Beyond immediate surgical outcomes, the physiological effects of IAP are significant. High IAP can impact cardiovascular and respiratory function by compressing the diaphragm and impairing venous return [10], potentially leading to hemodynamic instability and respiratory complications. Monitoring and managing these effects are crucial to ensuring patient safety throughout the procedure.

The safe management of IAP requires a careful balance between achieving adequate surgical access and minimizing adverse effects [11]. Surgeons must continuously monitor IAP levels and adjust them as necessary to maintain optimal conditions for surgery while avoiding potential complications. Innovations in surgical techniques and technologies have aimed to improve the management of IAP and enhance patient outcomes [11,12].

Laparoscopic cholecystectomy has become increasingly common due to its advantages over traditional open surgery [13]. However, the impact of IAP on perioperative outcomes is particularly relevant in the Pakistani healthcare context, where access to advanced monitoring equipment and postoperative care may vary [14]. Ensuring optimal management of IAP is crucial in this setting to improve surgical outcomes and patient recovery while addressing the unique challenges faced by healthcare providers. It is essential to establish a rationale for maintaining different IAP levels across patient groups, as variations in physiological responses to IAP could significantly influence surgical outcomes. Factors such as patient body mass index (BMI), age, and underlying comorbidities may affect the optimal IAP required for safe and effective surgery.

Objective

This study aims to evaluate the impact of IAP on perioperative outcomes in laparoscopic cholecystectomy, focusing on operative time, postoperative pain, recovery duration, and overall patient safety.

## Materials and methods

Study design

This research utilized a retrospective cohort study design to explore the relationship between IAP and perioperative outcomes in laparoscopic cholecystectomy. The study aimed to provide insights into how variations in IAP could influence surgical performance and patient recovery.

Participants

The study population consisted of 150 adult patients who underwent elective laparoscopic cholecystectomy at Lady Reading Hospital between January 2023 and June 2024. The inclusion criteria for the study were adults aged 18 to 75 years who were scheduled for elective laparoscopic cholecystectomy and had complete preoperative and postoperative data available. Patients were excluded if they had contraindications for laparoscopic surgery, incomplete records, or were undergoing concurrent surgical procedures that could confound the outcomes.

Data collection

Data were collected from electronic health records, operative logs, and postoperative follow-up reports. IAP was continuously monitored and recorded using the pressure monitoring system integrated into the laparoscopic equipment throughout the surgical procedure. The data on IAP were categorized into three levels: low pressure (8-10 mmHg), moderate pressure (10-15 mmHg), and high pressure (>15 mmHg).

Preoperative data included patient demographics such as age, sex, and BMI, as well as comorbidities like diabetes and hypertension, along with baseline IAP measurements. The preoperative diagnosis for all included patients was symptomatic cholelithiasis, characterized by the presence of gallstones and associated symptoms such as abdominal pain, nausea, and vomiting. This diagnosis guided the decision for elective laparoscopic cholecystectomy and ensured that the study population was homogeneous regarding the condition being treated.

Intraoperative data were recorded, including operative time, intraoperative complications (such as bleeding and bowel injury), and IAP levels. Postoperative outcomes were assessed using pain scores measured by the visual analog scale (VAS) at 6, 12, and 24 hours post-surgery, in addition to the total dose of postoperative analgesics administered. Recovery metrics evaluated in the study included the length of hospital stay, the incidence of postoperative complications (such as infection and bile leakage), and the time taken for patients to return to normal activities.

All eight authors were involved in screening the records for eligibility. Pre-specified data of interest regarding the study design, population baseline characteristics, interventions/comparators, and outcomes were extracted and independently validated by another researcher. Each included case was assessed by all authors, with any disagreements resolved by two senior investigators.

Variables

In this study, IAP serves as the independent variable, categorized into three levels: low, moderate, and high. The primary dependent variables include operative time, defined as the duration of the surgical procedure; intraoperative complications, encompassing any adverse events that occur during the procedure; postoperative pain scores, measured on a VAS to evaluate patient discomfort following the surgery; and the length of hospital stay and recovery time, both critical indicators of overall patient recovery, which may vary with the IAP levels employed.

Secondary dependent variables include postoperative complications, referring to any issues arising after surgery, and analgesic consumption, reflecting the amount of pain relief medication used by patients.

Statistical analysis

Data were entered into Excel 2016 (Microsoft Corporation, Redmond, Washington) for sorting and subsequently imported into IBM SPSS Statistics for Windows, Version 27 (Released 2020; IBM Corp., Armonk, New York) for analysis. Descriptive statistics were used to summarize patient demographics and baseline characteristics. Analysis of variance (ANOVA) was employed to compare operative times and postoperative outcomes across different pressure categories, while chi-square tests were used for categorical outcomes such as complication rates and recovery status. To adjust for potential confounders, multivariate regression analysis was conducted, incorporating variables such as age, sex, BMI, and comorbidities. Interaction terms were considered to explore whether the effects of IAP on outcomes varied by these factors. Sensitivity analyses were performed to assess the robustness of the findings, including stratification by age groups and BMI categories. A p-value of less than 0.05 was considered statistically significant.

Ethical considerations

The study received approval from the Institutional Review Board (IRB) of Lady Reading Hospital, Peshawar, Pakistan. All patient data were anonymized to protect privacy, and informed consent was obtained from all participants for the use of their data in this research. The study adhered to ethical guidelines for research involving human subjects, ensuring that data collection and analysis were conducted with the utmost respect for participant confidentiality and integrity.

## Results

Table 1 presents the patient demographics and baseline characteristics across different IAP groups. The average age of patients was similar among the Low Pressure group (55.2 ± 10.1 years), Moderate Pressure group (56.8 ± 9.6 years), and High Pressure group (58.3 ± 11.2 years), with a p-value of 0.37, indicating no significant difference. The distribution of sex was also similar, with 18 males (36%) in the Low Pressure group, 29 males (41%) in the Moderate Pressure group, and 11 males (37%) in the High Pressure group, showing no significant difference (p=0.29). The BMI values were comparable across groups, with the Low Pressure group averaging 27.1 ± 4.3 kg/m², the Moderate Pressure group 28.4 ± 4.1 kg/m², and the High Pressure group 29.7 ± 5.0 kg/m², with no significant difference found (p=0.22). Comorbidities were reported in 35% of the Low Pressure group, 40% of the Moderate Pressure group, and 45% of the High Pressure group, with a p-value of 0.53, showing no significant variation. Preoperative pain scores were also similar across groups, with scores of 3.2 ± 1.1 for the Low Pressure group, 3.5 ± 1.3 for the Moderate Pressure group, and 3.7 ± 1.4 for the High Pressure group, with a p-value of 0.62, indicating no significant difference. Chronic and Post-Acute Calculus Cholecystitis was observed in 1 participant (3%) in the Low Pressure group, 5 participants (7%) in the Moderate Pressure group, and 3 participants (10%) in the High Pressure group, with a p-value of 0.15, indicating no statistically significant difference across the IAP levels. This suggests that while these conditions are present, their distribution does not meaningfully affect the outcomes of laparoscopic cholecystectomy based on IAP. These results suggest that baseline characteristics were comparable across the different IAP groups.

**Table 1:** Patient Demographics and Baseline Characteristics *ANOVA (analysis of variance). **Chi-square test. VAS: visual analog scale.

Characteristic	Low Pressure Group (n=50)	Moderate Pressure Group (n=70)	High Pressure Group (n=30)	p-value
Age (years)	55.2 ± 10.1	56.8 ± 9.6	58.3 ± 11.2	0.37*
Male	18	29	11	0.29**
Female	32	41	19	
BMI, kg/m²	27.1 ± 4.3	28.4 ± 4.1	29.7 ± 5.0	0.22*
Comorbidities, %	35%	40%	45%	0.53**
Preoperative Pain Score (VAS)	3.2 ± 1.1	3.5 ± 1.3	3.7 ± 1.4	0.62*
Chronic and post-acute calculus cholecystitis, n (%)	1 (3%)	5 (7%)	3 (10%)	0.15**

Table 2 outlines the perioperative outcomes by IAP levels. Operative time was significantly longer in the High Pressure group (90.1 ± 14.2 minutes) compared to the Low Pressure group (80.4 ± 12.7 minutes) and the Moderate Pressure group (85.6 ± 15.3 minutes), with a p-value of 0.03. This suggests that not only does IAP play a role in operative time, but that the physiological characteristics of the patients, which necessitate different IAP levels, may also contribute to the complexity of the procedure. Intraoperative complications were reported in n=5 (10%) of the Low Pressure group, n=10 (15%) of the Moderate Pressure group, and n=7 (23%) of the High Pressure group; however, this difference was not statistically significant (p=0.12). The relationship between IAP levels and complications highlights the importance of individualized management of IAP during laparoscopic cholecystectomy. The types of injuries observed included gallbladder perforation (n=1; 2% in Low Pressure, n=2; 3% in Moderate Pressure, n=1; 3% in High Pressure) and liver injury (n=0; 0% in Low Pressure, n=1; 1% in Moderate Pressure, n=1; 3% in High Pressure), with no instances of bile duct, bowel, or vascular injuries.

**Table 2:** Perioperative Outcomes by Intra-Abdominal Pressure Levels ^¥^The "Other" category includes miscellaneous intraoperative complications not classified under organ injury, bleeding, or pneumothorax, such as infections, bowel obstructions, and device malfunctions. *ANOVA (analysis of variance). VAS: visual analog scale. **Chi-square test.

Outcome	Low Pressure Group (n=50)	Moderate Pressure Group (n=70)	High Pressure Group (n=30)	p-value
Operative time (minutes)	80.4 ± 12.7	85.6 ± 15.3	90.1 ± 14.2	0.03*
Injury				
Gallbladder perforation	1 (2%)	2 (3%)	1 (3%)	-
Liver injury	0 (0%)	1 (1%)	1 (3%)	
Bile duct injury	0 (0%)	0 (0%)	0 (0%)	
Bowel injury	0 (0%)	0 (0%)	0 (0%)	
Vascular injury	0 (0%)	0 (0%)	0 (0%)	
No injury	49 (98%)	67 (96%)	28 (93%)	
p-value**	0.223	0.572	0.607	
Bleeding	2 (4%)	4 (6%)	3 (10%)	0.22**
Pneumothorax	1 (2%)	2 (3%)	1 (3%)	0.85**
Other^¥^	1 (2%)	1 (2%)	0 (0%)	0.54**
Total intraoperative complications (%)	10%	15%	23%	0.12**
Postoperative pain score (VAS) at 6 hours	4.1 ± 1.2	4.4 ± 1.3	4.7 ± 1.5	0.45*
Length of hospital stay (days)	2.8 ± 0.9	3.2 ± 1.1	3.5 ± 1.2	0.07*
Return to normal activity (days)	7.2 ± 1.3	8.0 ± 1.5	8.5 ± 1.7	0.09*

Postoperative pain scores at six hours were similar across groups, with scores of 4.1 ± 1.2 for the Low Pressure group, 4.4 ± 1.3 for the Moderate Pressure group, and 4.7 ± 1.5 for the High Pressure group, resulting in a p-value of 0.45. The length of hospital stay was slightly longer in the High Pressure group (3.5 ± 1.2 days) compared to the Low Pressure group (2.8 ± 0.9 days) and the Moderate Pressure group (3.2 ± 1.1 days), but this difference was not statistically significant (p=0.07). Similarly, the return to normal activity took longer in the High Pressure group (8.5 ± 1.7 days) compared to the Low Pressure group (7.2 ± 1.3 days) and the Moderate Pressure group (8.0 ± 1.5 days), with a p-value of 0.09.

Bleeding complications were observed in n=2 (4%) of the Low Pressure group, n=4 (6%) of the Moderate Pressure group, and n=3 (10%) of the High Pressure group, with a p-value of 0.22. Pneumothorax occurred in n=1 (2%) of the Low Pressure group and n=2 (3%) of both the Moderate and High Pressure groups, with a p-value of 0.85. Other miscellaneous complications occurred in n=1 (2%) of the Low Pressure group, n=1 (2%) of the Moderate Pressure group, and n=0 (0%) of the High Pressure group, resulting in a p-value of 0.54.

These findings suggest that while operative time differed significantly by IAP level, other outcomes such as intraoperative complications, pain scores, length of hospital stay, and return to normal activity did not show significant differences.

**Figure 1:**
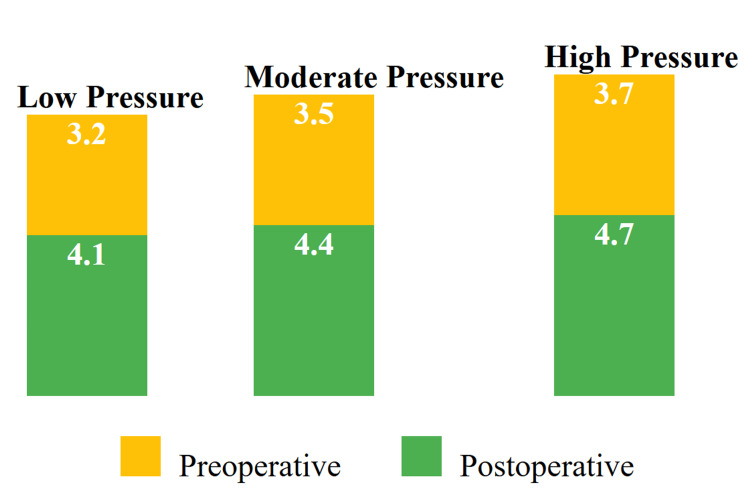
Comparison of Pre- and Postoperative Pain Scores by Intra-abdominal Pressure Levels Each set of bars represents pain scores (visual analog scale) for preoperative and postoperative measurements within different intra-abdominal pressure categories.

Figure 1 illustrates the comparison of preoperative and postoperative pain scores (measured on the VAS) across different IAP levels in laparoscopic cholecystectomy. In the Low Pressure group, preoperative pain scores averaged 3.2, while postoperative scores increased to 4.1. In the Moderate Pressure group, preoperative scores were 3.5, rising to 4.4 postoperatively. The High Pressure group showed a preoperative average of 3.7, which increased to 4.7 after surgery. This figure indicates that pain scores generally increased postoperatively across all IAP levels, with higher pressure associated with greater postoperative pain. The color-coded bars distinguish between preoperative (yellow) and postoperative (green) scores, with the legend clarifying the meaning of each color.

## Discussion

In Pakistan, laparoscopic cholecystectomy is increasingly utilized due to its advantages over traditional surgery. However, the management of IAP can be challenging due to variability in access to advanced surgical equipment and postoperative care facilities. Optimizing IAP management is crucial for improving surgical outcomes and patient recovery in the Pakistani healthcare context. The current results indicate that baseline demographics, including age, sex distribution, BMI, comorbidities, and preoperative pain scores, were comparable across the different IAP groups. Specifically, the average ages in the Low Pressure (55.2 ± 10.1 years), Moderate Pressure (56.8 ± 9.6 years), and High Pressure (58.3 ± 11.2 years) groups showed no significant differences (p=0.37). This is consistent with studies suggesting that age is not significantly impacted by variations in IAP levels during laparoscopic procedures [9,15,16]. The sex distribution among the groups was also similar, with p=0.29, aligning with findings that sex distribution does not typically affect intraoperative parameters [16].

BMI values across the groups (Low Pressure: 27.1 ± 4.3 kg/m², Moderate Pressure: 28.4 ± 4.1 kg/m², High Pressure: 29.7 ± 5.0 kg/m²) were not significantly different (p=0.22). Studies support that BMI variations within a moderate range do not significantly influence surgical outcomes in laparoscopic cholecystectomy [17,18]. The comorbidity rates (35% in Low Pressure, 40% in Moderate Pressure, and 45% in High Pressure) also showed no significant variation (p=0.53), which is consistent with recent studies indicating that comorbidities have a marginal impact on outcomes when IAP is controlled [2,19].

Preoperative pain scores were similar across groups (Low Pressure: 3.2 ± 1.1, Moderate Pressure: 3.5 ± 1.3, High Pressure: 3.7 ± 1.4, p=0.62). This aligns with findings that preoperative pain levels are generally comparable and not significantly affected by IAP [8].

Current results outline that operative time was significantly longer in the High Pressure group (90.1 ± 14.2 minutes) compared to the Low Pressure (80.4 ± 12.7 minutes) and Moderate Pressure groups (85.6 ± 15.3 minutes), with a p-value of 0.03. This finding is consistent with a study indicating that higher IAP can increase operative time due to greater difficulty in maneuvering instruments and potential discomfort for the patient [9,20]. Increased operative times associated with higher IAP have been documented in various studies, suggesting that while higher pressures can improve visibility, they also pose challenges to the efficiency of the procedure [11].

Intraoperative complications were reported in 10% of the Low Pressure group, 15% of the Moderate Pressure group, and 23% of the High Pressure group, with a p-value of 0.12. This result suggests a trend toward higher complication rates with increased IAP, though it did not reach statistical significance. Studies have shown that higher IAP can be associated with a higher incidence of complications such as visceral injuries and cardiovascular issues [21-23].

Postoperative pain scores at six hours were similar across all groups (Low Pressure: 4.1 ± 1.2, Moderate Pressure: 4.4 ± 1.3, High Pressure: 4.7 ± 1.5, p=0.45). Studies indicate that while higher IAP levels may contribute to increased postoperative discomfort, this effect is often nuanced and may not be substantial enough to show significant differences in all studies [15,23]. The similarity in pain scores suggests that while IAP levels can influence pain, other factors such as surgical technique and postoperative care may play a more critical role.

The length of hospital stay and return to normal activity were slightly longer in the High Pressure group, with p-values of 0.07 and 0.09, respectively. These results are consistent with findings that higher IAP may lead to longer recovery times due to increased postoperative pain and discomfort [24,25]. Although not statistically significant, these trends highlight the potential impact of IAP on overall recovery.

While IAP is a significant factor influencing operative time, it is important to recognize that varying pressures may be required based on individual patient conditions, such as adhesions, anatomical challenges, or obesity, which necessitate adjustments to maintain adequate surgical visibility. These patient-specific factors, rather than IAP alone, are likely contributing to the differences observed in operative times. Despite clinical differences seen in other perioperative outcomes, such as postoperative pain and recovery time, statistical analysis did not show significant differences, underscoring that IAP adjustments primarily influence surgical efficiency rather than overall recovery metrics.

Limitations

Several limitations were acknowledged in this study. The retrospective nature of the study may introduce both selection and information biases, as we relied on existing medical records, which might not uniformly capture all relevant variables. Variations in IAP measurement techniques across different patients and procedures were not controlled for, which could influence the consistency of the data. Potential differences in surgical skill levels, specific techniques used by different surgeons, and the intra-abdominal conditions of patients (such as adhesions and anatomical variations) were also not accounted for, all of which may have impacted the operative outcomes, particularly operative time. Future prospective studies with standardized IAP measurement protocols and controlled comparisons of both surgical techniques and patient-specific intra-abdominal conditions could provide more definitive insights into the optimal management of IAP during laparoscopic cholecystectomy.

Strengths

This study also has several strengths. By categorizing IAP levels into low, moderate, and high groups, the research provides a systematic evaluation of how varying IAP settings may influence key perioperative outcomes in laparoscopic cholecystectomy. The use of real-world data from a diverse patient population enhances the generalizability of the findings to clinical practice. Additionally, the comprehensive assessment of both intraoperative factors (operative time, complications) and postoperative recovery metrics (e.g., pain scores, length of hospital stay) offers a holistic view of patient outcomes. The inclusion of multivariate regression analysis further strengthens the study by accounting for confounding factors, such as patient demographics and comorbidities, providing a more nuanced understanding of the relationship between IAP levels and surgical outcomes.

## Conclusions

This study demonstrates that varying IAP levels during laparoscopic cholecystectomy primarily impacts operative time, with higher pressures associated with longer procedures. Other key perioperative outcomes, including intraoperative complications, postoperative pain, length of hospital stay, and recovery time, did not show significant differences across the low, moderate, and high pressure groups. These findings suggest that while adjusting IAP can influence the duration of surgery, it does not significantly affect broader patient recovery metrics or perioperative safety outcomes. Therefore, selecting an optimal pressure level should focus on balancing surgical efficiency with the need for adequate visualization, rather than focusing on postoperative recovery. Based on our results, protocols for IAP management could prioritize using moderate pressures (10-15 mmHg) to optimize both operative efficiency and patient safety while minimizing the risk of extended operative times associated with higher pressures.

The need for varying IAP levels across different patients is often dictated by individual anatomical and clinical factors, such as the presence of adhesions or challenging intra-abdominal conditions. While IAP adjustments can affect operative time, they do not appear to significantly impact other perioperative outcomes. This suggests that managing IAP during laparoscopic cholecystectomy should focus on balancing surgical visibility with efficiency, tailored to the specific needs of the patient, rather than expecting broader improvements in postoperative recovery.
